# Coping with an Acute Psychosocial Challenge: Behavioral and Physiological Responses in Young Women

**DOI:** 10.1371/journal.pone.0114640

**Published:** 2014-12-09

**Authors:** Carolina Villada, Vanesa Hidalgo, Mercedes Almela, Francesca Mastorci, Andrea Sgoifo, Alicia Salvador

**Affiliations:** 1 Laboratory of Social Cognitive Neuroscience, University of Valencia, Valencia, Spain; 2 Extreme Centre, Institute of Life Sciences, Scuola Superiore Sant’Anna, Pisa, Italy; 3 Stress Physiology Laboratory, Department of Neuroscience, University of Parma, Parma, Italy; Sapienza University of Rome, Italy

## Abstract

Despite the relevance of behavior in understanding individual differences in the strategies used to cope with stressors, behavioral responses and their relationships with psychobiological changes have received little attention. In this study on young women, we aimed at analyzing the associations among different components of the stress response and behavioral coping using a laboratory psychosocial stressor. The Ethological Coding System for Interviews, as well as neuroendocrine, autonomic and mood parameters, were used to measure the stress response in 34 young women (17 free-cycling women in their early follicular phase and 17 oral contraceptive users) subjected to the Trier Social Stress Test (TSST) and a control condition in a crossover design. No significant differences in cardiac autonomic, negative mood and anxiety responses to the stressor were observed between the two groups of women. However, women in the follicular phase showed a higher cortisol response and a larger decrease in positive mood during the social stress episode, as well as greater anxiety overall. Interestingly, the amount of displacement behavior exhibited during the speaking task of the TSST was positively related to anxiety levels preceding the test, but negatively related to baseline and stress response values of heart rate. Moreover, the amount of submissive behavior was negatively related to basal cortisol levels. Finally, eye contact and low-aggressiveness behaviors were associated with a worsening in mood. Overall, these findings emphasize the close relationship between coping behavior and psychobiological reactions, as well as the role of individual variations in the strategy of coping with a psychosocial stressor.

## Introduction

In recent years, growing recognition of the effect of stress on health has led to intense research on individual differences in coping with an environmental challenge, mainly employing controlled laboratory stressors. Among them, the Trier Social Stress Test (TSST) has been used extensively as a psychosocial stress paradigm [1]. Due to its uncontrollability and evaluative-threat properties [2], it is able to provoke clear adrenocortical, autonomic and mood changes [3]–[6]. A large amount of attention has been focused on these psychophysiological mediators of the “stress-health” relationship, while also trying to explain the considerable individual variations in the stress response and vulnerability [7].

In young people, most of the research on acute stress was initially carried out in men, but it has been increasingly accepted that sex hormones, or their absence, play a role in the response to stress [8]. The first large impact study employing the TSST to determine the influence of sex steroids on the stress response found that men and luteal women showed a similar cortisol response, which was higher than that of women in the follicular phase and oral contraceptive users (OC users), with the latter two groups exhibiting similar cortisol, cardiovascular and mood responses [6]. Since then, numerous studies have been carried out on women, but only a few studies have included women in menstrual cycle phases other than the luteal phase [9]–[11] or women taking oral contraceptives [12], [13]. Overall, these studies have reported no sex differences in heart rate, although lower cortisol responses and larger increases in negative mood have been found in free-cycling women compared to men [10], [11]. In addition, women taking oral contraceptives have generally shown a blunted cortisol response compared to free-cycling women [14], specifically in the luteal phase [13]. Most studies have shown that these differences are smaller when the follicular phase is compared to the use of oral contraceptives. In sum, there is some evidence suggesting that women’s hormonal status affects the hypothalamic-pituitary-adrenal (HPA) axis and autonomic nervous system (ANS) responses to laboratory stressors.

Apart from their physiological responses and subjective perceptions, when people face a psychosocial stressor they adopt a set of behavioral strategies that play an important role in coping with the situation. The ethological approach is a valuable tool for studying individual differences in behavioral coping strategies, and it can provide further insight into individual responsivity to a psychosocial stressor [15], [16]. Physiological and behavioral stress responses have been grouped in the so-called “active/proactive” or “passive/reactive” coping styles [17], [18], whereas cognitive dimensions have been emphasized in humans [19]. Unlike the fight-or-flight response to stress [20], the tend-and-befriend theory described by Taylor (2000) [21] proposes that women reveal an adaptive reaction to stress that is more related to affiliative behaviors, which promote social interactions. Therefore, studying behavioral stress responses in women could help providing insight on the relationship between behavioral style of coping and physiological stress responsivity.

To the best of our knowledge, only a few studies have been carried out in healthy young people using an ethological approach to obtain additional information about the interrelationships among different psychophysiological markers of stress responsivity. Sgoifo et al. (2003) compared men and women (undifferentiated phase of the cycle) faced with a social stressor (interview) [22]. A few years later, using the same experimental approach, Pico-Alfonso et al. (2007) compared women in the follicular and ovulatory phases [15]. These two studies found several interesting relationships between psychophysiological changes and a number of behavioral patterns displayed during the stressful task. For example, escape (flight) behavior (e.g. look away or look down: social contact is temporarily broken off by disengaging from any interaction) was negatively related to cortisol response [22], whereas cardiac activation was positively associated with submissive behavior (e.g mouth corners back or lips in) and negatively with displacement behavior [15]. Recently, it has been suggested that displacement behaviors (a set of behavioral patterns that consists of movements which are focused on one’s own body such as hand-face or scratch) would regulate the cardiovascular stress response in men but not in luteal women [23], whereas the relationship between behavior and physiological stress response in women seems to be modulated mostly by the subjective perception of stress [24].

The purpose of the present study was to analyze the behaviors displayed during the TSST and their relationships with (i) the responses of the two most frequently employed biomarkers of acute stress response (cortisol and heart rate) and (ii) the mood experienced by women in the follicular phase or taking oral contraceptives. These two groups, which are both characterized by low oestrogen concentrations, were shown to have similar cortisol, heart rate, and mood responses to the TSST [6], [14].

At first we wanted to assess whether the psychophysiological response to stress was different between the two groups of women. Although, according to the available evidence, dramatic differences were not likely to be found among follicular women and those making use of contraceptives, we believe that such a comparison could provide an additional piece of information to the available literature. In addition, we hypothesized that acute physiological stress reactivity is modulated by the behavioral patterns of response exhibited during the speech task. More specifically, we anticipated that the intensity of cardiovascular and cortisol responses are associated with the amount of submissive and escape behaviors, which are commonly associated with a passive coping style [15], [22]. We also expected to confirm previous findings suggesting that displacement behavior is tightly linked to individual physiology and anxiety [23], [24]; in other words, we anticipated a positive relationship between displacement behavior and pre-stress levels of anxiety, and negative relationships with baseline and stress response values of heart rate [15], [22], [23], [24]. Moreover, given that these groups of women have never been studied from an ethological perspective, we aimed at exploring the relationships among behavioral and psychophysiological stress responses also split by group, to provide new information to the literature considering the menstrual cycle phase and the use of oral contraceptives.

Finally, we aimed at exploring possible associations between coping strategies (as detected via the evaluation of self-reports) and non-verbal behavior displayed during the speech test, which have never been reported in the literature so far.

## Methods

### Ethics Statement

The study was conducted in accordance with the Declaration of Helsinki, and the protocol and conduct were approved by the University of Valencia Ethics Research Committee. All the participants received verbal and written information about the study and signed an informed consent form.

### Participants

A total of 107 female volunteers, recruited through advertisements in the university campus, were interviewed and completed a questionnaire to find out whether they met the study prerequisites. The criteria for exclusion were: smoking more than five cigarettes a day, alcohol or other drug abuse, visual or hearing problems, presence of a cardiovascular, endocrine, neurological or psychiatric disease, and the presence of a stressful life event during the last year. Participants were excluded if they were using any medication directly related to cardiac, emotional or cognitive function, or one that was able to influence hormonal levels, such as glucocorticoids or β-blockers.

Finally, 34 women (between 18 and 29 years old) participated in two sessions in a counterbalanced order. All of them were nulliparous with no gynecological problems. Half were free-cycling, with regular menstrual cycle lengths of between 24 and 36 days. The follicular phase was chosen because it is the most reliable in the absence of sex steroid analyses; in addition, we selected the early follicular phase (2–5 days after the beginning of the menstruation) because this period has been studied less in this research field. The other half of the women had been taking oral contraceptives (monophasic formulas) for at least 6 months. Socio-demographic data are presented in Table 1. Most of the participants (94%) were college students from different fields (psychology, medicine, occupational sciences, among others), and none of the participants received academic or economic compensation for their participation.

**Table 1:** Demographic and anthropometric characteristics of total sample and subgroups (Mean scores ± SEM) of age, Body Mass Index, Subjective Socioeconomic Status (Subjective SES scale [44]) and physical exercise.

	OCusers	FollicularW	Total Sample
	(n = 17)	(n = 17)	(n = 34)
BMI (Body Mass Index) Kg/m2	21.54±0.68	21.85±0.69	21.70±0.48
Age (years)	20±0.707	22.59±0.72	21.29±0.545
SES (scores 1–10)	6.47±0.273	6.12±0.225	6.29±0.177
Physical Exercise (scores 0–7)	3.41±0.59	3.63±0.54	3.52±0.39

Participants meeting the criteria were contacted by telephone and asked to attend experimental sessions that took place in a laboratory at the Faculty of Psychology (University of Valencia, Spain). Before each session, participants were asked to maintain their general habits, sleep as long as usual, refrain from heavy activity the day before the session, and not consume alcohol since the night before the session. Additionally, they were instructed to only drink water, and not eat or take any stimulants such as coffee, cola, caffeine, tea or chocolate, two hours prior to the session.

### Study protocol

This study employed a within-subject design with two completely randomized and counterbalanced conditions in two separate sessions: a stress condition and a control condition.

In both conditions, each session lasted approximately 1 hour, and they were always held between 16.00 and 19.00 hours. Each participant started her two sessions at the same time of the day. In the first session, upon arrival at the laboratory, the participants’ weight and height were measured, and the experimenter checked whether they had followed the instructions given previously. In the last part of this first session, all the participants completed the personality questionnaires.

*Stress Condition.* To produce a stress response, the participants were subjected to the Trier Social Stress Test (TSST). The stress task consisted of 5 min of free speech (job interview) and a 5 min arithmetic task (see Kirschbaum et al. 1993, for details) [1], and it was performed in front of a committee composed of a man and a woman (both university teachers in the psychology department). The participants remained standing at a distance of 1.5 meters from the committee. Additionally, a video camera and a microphone were clearly visible, and the speech task was video recorded.

The protocol started with a habituation phase of 15 min to allow the participants to adapt to the laboratory setting. During this phase, the participants remained seated. Five minutes after this phase started, baseline measures were obtained for anxiety (Stai-S) and mood (PANAS). After the habituation phase, the introduction phase started (duration 3 min). In this phase, participants were informed about the procedure for the stress task. They received the instructions in front of the committee in the same room where the task took place. Next, the participants had 10 minutes to prepare for the task at hand in the first room. During this period, they provided a saliva sample (−5 min pre-stress).

Following the preparation phase, the stress test was carried out. Subjects had 20 minutes to recover after the 10-min stress task; during this period, they again answered the two questionnaires (Stai-S and PANAS) and provided the second saliva sample (+15 min post-stress). The participants provided the last saliva sample 25 minutes later (+40 min post-stress).

*Control Condition.* The control condition was similar to the experimental condition, except that the stressful task was replaced by a control task. This task was designed to be similar to the stress task in mental workload and global physical activity, but without the main components capable of provoking stress, such as evaluative threat and uncontrollability [2].

The control task consisted of 5 minutes of reading aloud and 5 minutes of counting, but not in front of an audience. In the preparation phase, the participants did not prepare for their task; instead, they read a book with neutral content. The times for the saliva samples, the questionnaires, and the phase durations were the same as those described for the stress condition.

### Biochemical analyses

#### Cortisol

Participants provided three saliva samples of 3 ml each in plastic vials. They took approximately 5 minutes to fill the vial. The samples were frozen at −80°C until the analyses were performed. The samples were analyzed by a competitive solid phase radioimmunoassay (tube coated), using the commercial kit Coat-A-Count C (DPC, Siemens Medical Solutions Diagnostics, Los Angeles, CA, USA). Assay sensitivity was 0.5 ng/mL. The findings are expressed in nanomolar units (nmol/L). For each participant, all the samples were analyzed in the same trial. The within and inter assay variation coefficients were all below 8%.

### Heart Rate measurements

#### Heart Rate (HR)

HR was continuously recorded in the experimental and control conditions using the heart rate monitor Suunto T6 (Suunto Oy, Vantaa, Finland), which consists of a chest belt for detection and transmission of the heartbeat and a “watch” for data collection and storage [25], [26]. Heartbeat detection is performed with an accuracy of 1 ms, every heartbeat is transmitted and stored in the flash memory of the watch. The recording periods when participants were walking from one room to another were removed, and only the 5 central minutes of each phase - namely (from −20 to −15 min for baseline, from −6 to −1 min for preparation, from 0 to +5 for speech and from +15 to +20 for recovery) - were used to calculate the participants’ average heart rate values. After eliminating the artifacts, the HR mean for each phase was computed. HR artifacts and HR analysis were performed with Kubios software (Biomedical Signal Analysis Group, University of Kuopio, Finland).

### Coping Strategies

The dispositional version of the COPE Inventory is a theoretically driven self-report questionnaire that addresses different ways of coping [27]. Subjects must indicate what they *generally* do and feel when experiencing stress. Items are rated on a 4-point scale, ranging from 1 (*I don’t usually do this at all*) to 4 (*I usually do this a lot*). We employed the Spanish version of the long form, which consists of 60 items from 15 subscales (such as Planning, Seeking Instrumental Support, Suppression of Competing Activities, Restraint Coping, Venting of Emotions, among others). With a second-order factor analysis, they can be grouped in five basic coping domains: behavioral, cognitive and emotional coping measures (active coping), and behavioral and cognitive avoidance (passive coping). The Spanish version of the scale had Cronbach’s alphas ranging from 0.78 to 0.92 [28].

### Psychological assessment

#### Anxiety

To assess state anxiety, the Spanish version of the State Anxiety Inventory was used (STAI form E) [29]. It consists of 20 phrases (e.g. ‘*I feel at ease’,* ‘*I feel upset’*), with a 4-point Likert scale ranging from 0 (not at all) to 3 (extremely) to evaluate how the participants felt at the moment they gave the answers. The Spanish version of the scale had Cronbach’s alphas ranging from 0.90 to 0.93 [30].

#### Mood

The mood was evaluated by the Spanish version [31] of the PANAS (Positive and Negative Affect Schedule) [32]. This 20-item questionnaire assesses mood according to two dimensions: positive affect (PA: *interested*, *excited*, *strong*, *enthusiastic*, etc.) and negative affect (NA: *distressed, upset, guilty, scared*, etc.), with 10 items measuring each state. Participants were asked to complete the questionnaire based on how they felt at that particular moment. They responded using a 5-point Likert scale ranging from 1 (not at all) to 5 (extremely). Sandin et al. (1999) [31] reported a high internal consistency for the Spanish version, with Cronbach’s alphas for PA ranging from 0.87 to 0.89, and for NA from 0.89 to 0.91.

### Ethological analysis

The participants’ behavior during the speech task of the TSST was quantified by means of the Ethological Coding System for Interviews (ECSI) [16]. The interview was videotaped with a camera adjusted so that the subject’s face and trunk were in full view. Subsequently, behavioral assessment was carried out according to Troisi and colleagues [15], [16], [22]. This version of the ECSI includes 32 different patterns, mostly facial expressions and hand movements. The ECSI was specifically designed to measure non-verbal behavior during stress interviews by combining behavior patterns described in published human ethograms [16]. The 32 behavioral patterns were then grouped in seven behavioral categories, each reflecting a different aspect of the subject’s emotional and social attitude [16], namely: (1) eye contact; (2) affiliation; (3) submission; (4) flight; (5) assertion; (6) gesture; (7) displacement. The score of a given behavioral category was expressed as the sum of the percentages of all the behavioral patterns belonging to it [15].

### Statistical analyses

One-way ANOVAs were used to analyze differences between groups in the demographic/anthropometric variables and behavioral patterns. We employed Group (follicular women vs. OC users) as a between-subject factor and Condition (stress vs. control) as a within-subject factor. ANOVAs for repeated measures were used to assess the effects on mood, anxiety, cortisol and heart rate. For the mood and anxiety analyses, we added a within-subject factor: pre and post task. For the HR analyses, we added Time (−20, −5, +5, and +15 min) as a within-subject factor; for the cortisol analyses, we also added Time (−5, +15, +40 min) as a within-subject factor. Additionally, cortisol and stress-induced HR reactivity were also quantified as the area under the response time curve to ground (AUC_G_) [33]; finally, for anxiety and mood changes, the differences between post-task and pre-task scores (Delta) were calculated.

We checked for order effects (whether the stress or control condition was first) by using an ANOVA for repeated measures, which did not reveal any effect of order (all *p*>0.18).

Pearson’s correlations (two-tailed) were calculated in order to assess whether the physiological (basal levels and AUC_G_) and psychological values (basal levels and Deltas) were related to the behavioral patterns. For the analysis of ethological data, six subjects were removed: 3 OC users due to technical problems with the images analyzed, and 3 women in the follicular phase on the basis of the *p*>0.001 criteria for Mahalanobis distances for eye contact behavior. We used Greenhouse-Geisser when the requirement of sphericity in the ANOVA for repeated measures was violated. Post hoc planned comparisons were performed using the Bonferroni adjustments for the *p*-values. All *p*-values reported are two-tailed, and the level of significance was marked at <0.05. When not otherwise specified, results shown are means ± standard error of means (SEM). We used SPSS 19.0 to perform the statistical analyses.

## Results

### Cortisol

ANOVAs for repeated measures showed significant effects of Condition, F(1,32) = 7.043, *p* = .012, η^2^_p_ = .108, Time, F(1.362,43.569) = 5.387, *p* = .016, η^2^_p_ = .144, and the Condition×Time interaction, F(1.34,42.893) = 21.867, *p*<.001, η^2^_p_ = .406. Post-hoc analyses revealed that there were no baseline differences between conditions, *p* = .265, whereas after the task (+15 min and +40 min time points), differences between conditions were found (both *p*<.01).

**Figure 1:**
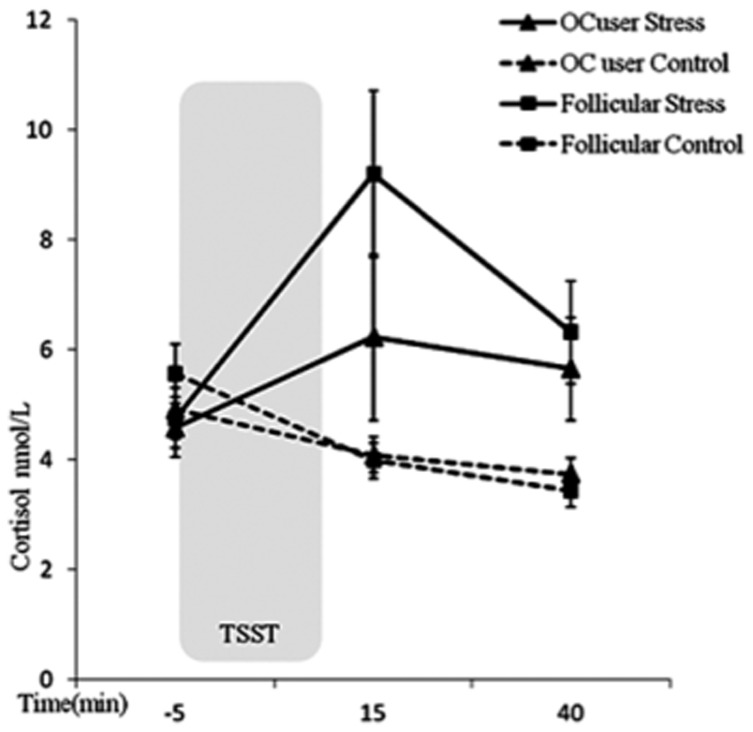
Mean values ±SEM salivary cortisol response (nmol/L) during the stress and control conditions by group (*p<0.05).

A marginal Condition×Time×Group interaction was found, F(1.34, 42.893) = 3.65, *p* = .051, η^2^_p_ = .101. Fig. 1 shows no statistically significant differences between groups (follicular and OC users) at any time point within each condition (stress and control) (all *p*>.176). Women in the early follicular phase showed significantly higher cortisol levels in the stress condition than in the control condition at +15 min and +40 min samples (all *p<*.01). OC users did show a similar difference in the time course of cortisol levels between the stress and control condition, although statistical significance was not reached (+15 min, *p* = .171; +40 min, *p* = .051).

### Heart Rate (HR)

ANOVA for repeated measures revealed a significant effect of the Condition×Time interaction, F(2.236, 67.091) = 16.856, *p*<.001, η^2^_p_ = .228. No differences between conditions (stress vs. control) were found in the baseline [stress condition: (83.97±1.34) vs. control condition (85.43±1.69)] and recovery periods [stress condition: (78.94±1.62) vs. control condition (77.16±1.53)] (both *p*>0.1), but there were higher HR values in the stress condition than in the control condition in the two key periods of the TSST: Preparation [stress condition: (90.31±1.89) vs. control condition (83.25±1.6)]and Speech [stress condition: (101.89±2.58) vs. control condition (93.29±1.77)] (both *p*<.01). No significant effects of Group or other interactions were found.

### State Anxiety

A repeated-measures ANOVA of the STAI scores revealed significant effects of Condition, F(1, 32) = 25.826, *p*<.001, η^2^_p_ = .447, Time, F(1, 32) = 21.268, *p*<.001, η^2^_p_ = .399, and the Condition×Time interaction, F(1, 32) = 31.511, *p*<.001, η^2^_p_ = .526. Post hoc analyses revealed that anxiety increased significantly after the stress condition (*p*<.001) and slightly decreased after the control condition (*p* = .076). A main effect of Group was also observed, F(1, 32) = 5.233, *p* = .029, η^2^_p_ = .141: overall, women in their follicular phase obtained significantly higher scores on anxiety than the OC users (Mean±sem for follicular women: 20±1.4; for OC users: 15.4±1.41).

### Mood

**Figure 2:**
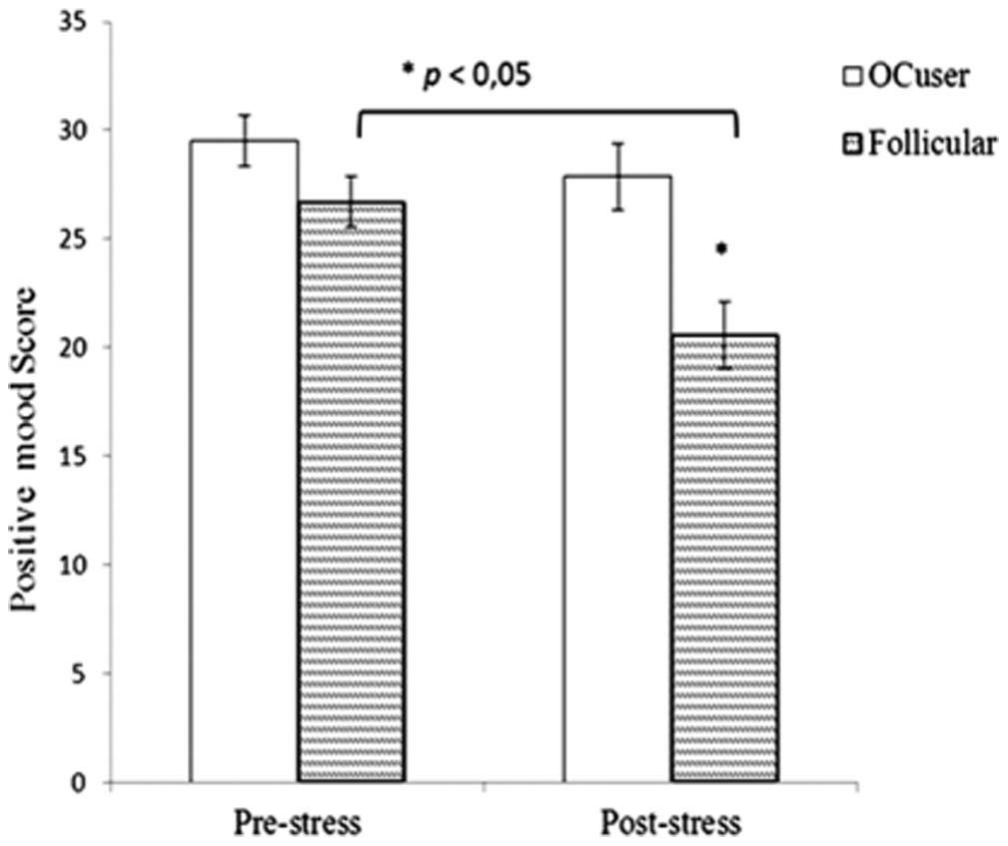
Mean values ± SEM of positive mood scores during the stress condition by group (*p<.01).

For positive mood, significant effects of Time, F(1, 32) = 10.242, *p* = .003, η^2^_p_ = .242, Group, F(1,32) = 11.865, *p* = .002, η^2^_p_ = .27, and the Condition×Time×Group interaction were found, F(1, 32) = 4.774, *p* = .036, η^2^_p_ = .13. Women in their follicular phase showed a decrease in positive mood after the stress task, compared to their pre-stress scores (*p*<.001), whereas OC users did not show stress-related changes in this parameter (*p* = .17). Significant, between-group differences were observed after the TSST, with follicular women showing lower positive mood than OC users (*p* = .002) (see Fig. 2).

For negative mood, significant effects of Condition, F(1, 32) = 24.308, *p*<.001, η^2^_p_ = .432), Time, F(1, 32) = 11.724, *p* = .002, η^2^_p_ = .268) and the Condition × Time interaction were found, F(1, 32) = 40,431, *p*<.001, η^2^_p_ = .558. Overall, negative mood increased after the stress task (*p*<.001) and decreased after the control task (*p*<.001). No significant effects of Group or interactions were found (*p*>.1). [Stress condition: pre-task (12.9±0.4) vs. post-task (18.1±1.04); control condition: pre-task (13.8±0.6) vs. post-task (12.1±0.4)].

### Ethological data (ECSI)

Table 2 summarizes the behavioral response exhibited by the two groups of women (follicular and OC users) during the speech task, each behavioral category being quantified as cumulative percentages. The overall sample was characterized by higher values of flight and affiliation behaviors compared to the other behavioral categories analyzed.

**Table 2:** Total sum of the behavioral patterns’ values of each behavioral category (% mean ± SEM) displayed during speech (ECSI).

Ethological Data	OCusers (n = 14)	FollicularW (n = 14)	Total Sample (n = 28)
**EYE CONTACT**	**83.21±3.03**	**93.57±1.84**	88.39±2.01
FLIGHT	120±8.25	134.64±7.27	127.32±5.57
SUBMISSION	17.85±3.73	22.14±4.53	20±2.90
AFFILIATION	116.07±10.99	122.14±14.63	119.11±9.0
GESTURE	17.14±7.99	2.85±2.85	10±4.38
DISPLACEMENT	76.42±8.91	82.14±13.18	79.28±7.82
**ASSERTION**	**9.64±3.03**	**28.92±5.86**	19.28±3.73

(In bold are the categories with significant between-group differences, both *p* = 0.007).

Women in their follicular phase displayed higher percentages compared to OC users, for all the behavioral categories, except for Gesture (Table 2). However, statistically significant differences between groups only appeared of them: Eye Contact, F(1,27) = 8.482, *p* = .007, and Assertion, F(1,27) = 8.534, *p* = .007 (Table 2).

### Relationships among Behavioral and Psychobiological responses to the TSST

As the differences between groups in the stress response (cortisol, heart rate and subjective stress response) were rather sporadic, and in order to facilitate potential comparisons with results of previous studies [15], [22], correlations were performed considering the two female groups as a whole.

The amount of displacement behavior exhibited during the speech task was negatively related to the basal levels and AUC_G_ of the HR (*r* = −.545, *p* = .004; *r* = −.492, *p* = .013, respectively) and positively related to the degree of anxiety experienced before the TSST (*r* = .448, *p* = .017). Furthermore, the amount of eye contact correlated negatively with negative mood before the stress task (*r* = −.375, *p* = .05). The expression of behavioral patterns included in the assertion category (low-aggressiveness) was positively related to _Delta_negative mood (*r* = .415, *p* = .028), and the anxiety reaction to stress (*r* = .378, *p* = .047), but inversely to _Delta_positive mood (*r* = −.570, *p* = .002).

Finally, submissive behaviors during the speech were negatively related to basal cortisol levels (*r* = −.402, *p* = .034), but did not reach significance with the AUC_G_ values of this hormone (*r* = −.320, *p* = .09).

Subsequently, given that these groups did show some differences in cortisol, anxiety and mood, and with the aim of providing new information on the relationship between neuroendocrine and behavioral stress responsivity, correlation analyses were also performed split by group.

We found out that the relationships previously found for the study group as a whole were confirmed for the group of women in the follicular phase (see table 3). In addition, OC users showed: (i) positive associations of both Affiliation and Flight with the basal levels and the AUCg of cortisol; (ii) negative relationship between anxiety and negative mood (see table 4).

**Table 3:** Pearson correlations between psychophysiological basal levels, AUCg of cortisol and HR, anxiety and mood reactivity with the behavioral patterns performed during the speaking task of the TSST (data from women in the follicular group).

Follicular		BL_Cort	AUCg	BL_HR	AUCg	STAIpre	Delta	Delta
W			Cort		HR		STAI	mood(+)
	SUBMISSION	r = −0,559	r = −0,479	ns	ns	ns	ns	
		*p* = .038	*p* = .083					
	DISPLACEMENT	ns	ns	r = −0,692	r = −0,585	r = 0,56	ns	
				*p* = .009	*p* = .036	*p* = .037		
	ASSERTION	r = 0,532	ns	ns	ns	ns	r = 0,667	r = −0,549
		*p* = .05					*p* = .009	*p* = .042

**Table 4:** Pearson correlations between psychophysiological basal levels and AUCg of cortisol, anxiety and mood reactivity with the behavioral patterns performed during the speaking task of the TSST (data from OCusers).

OCusers		BL_Cort	AUCg Cort	Delta STAI	Delta mood (−)
	FLIGHT	ns	ns	r = 0,721	r = 0,662
				*p* = .004	*p* = .01
	AFFILIATION	r = 0,718	r = 0,584	ns	ns
		*p* = .004	*p* = .028	ns	ns

### Coping styles (COPE)

**Figure 3:**
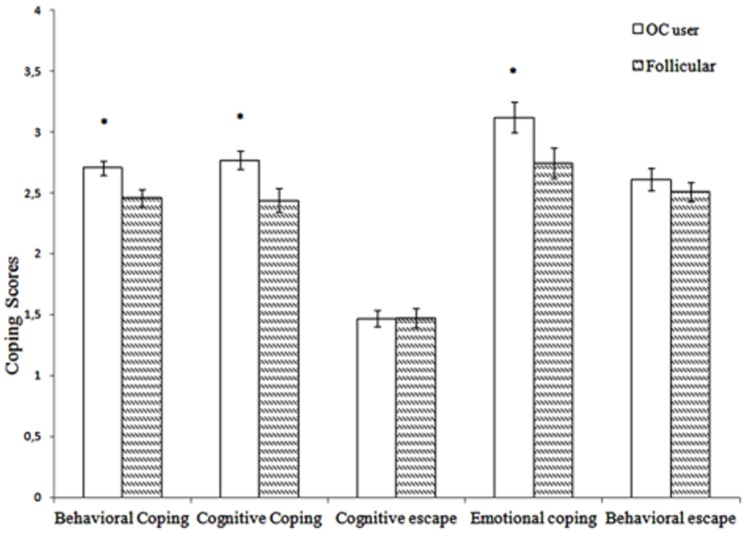
Mean values ± SEM of the five second-order factors of the COPE questionnaire that represents active coping (Behavioral coping, Cognitive coping, and Emotional coping) and passive coping (Cognitive escape and Behavioral escape) (*p<.05).

A one-way ANOVA with a second-order factor analysis of the COPE questionnaire showed a main effect of Group for Factor I_(behavioral coping)_, F(1, 32) = 7.036, *p* = .012, η^2^_p_ = .180, Factor II_(cognitive coping)_, F(1, 32) = 7.298, *p* = .011, η^2^_p_ = .186, and Factor IV_(emotional coping)_, F(1, 32) = 4.366, *p* = .045, η^2^_p_ = .12. Follicular women had lower scores than their OC counterparts on all these factors (Fig. 3), reflecting differences in the dimensions of active coping. No differences between groups were found in dimensions that indicate passive coping (all *p*>.397).

A few significant correlations were found between the basic coping styles and some behavioral categories. Specifically, active coping correlated negatively with assertion and eye contact (*r* = −.450, *p* = .016; *r* = −.461, *p* = .014, respectively), and marginally with the amount of affiliation exhibited (*r* = .362, *p* = .058).

## Discussion

The present study investigated the subjects’ physiological response, subjective anxiety and mood, as well as their nonverbal behavior, while undergoing the Trier Social Stress Test (TSST). This study provides new insights on the relationship between the patterns of behavior displayed during a public speaking task and a number of psychobiological stress-related changes. To this purpose, women in their follicular phase and women taking oral contraceptives were exposed to an experimental condition and a control condition in a within-subject design.

Our results confirmed that the TSST provoked significant changes in cortisol, heart rate, anxiety and mood, in accordance with other studies that have examined both psychological and physiological responses to this type of laboratory stressor [10], [14], [34], [35], [36]. As originally hypothesized, stress-induced cortisol and heart rate increases were modest but significant, and no major differences were found in the physiological stress response of oral contraceptive users compared to women in their follicular phase, these results largely resembling those reported by Kirschbaum et al. (1999) [6]. The blunted cortisol response of OC users is also in accordance with a previous study on adolescent women, where another psychosocial stress paradigm (Groningen Social Stress Test) was employed [37].

We observed a marked decrease in positive mood following the stress episode only in women in their follicular phase, suggesting that they are more sensitive to stress-induced changes in mood. Interestingly no significant differences were found on this regard in two previous studies that compared free-cycling women with OC users [14], [37]. Recently, however, an association between psychological complaints and the follicular phase of the menstrual cycle has been reported [38]. It is worthwhile to note that women in our study were tested in the earliest follicular phase, which means that their stress-induced, short-term psychological consequences could be due to the discomfort of menstruation symptoms themselves. Although this discomfort was not objectively measured in this study, we found that women in the early follicular phase also showed higher scores on overall anxiety, and also displayed a larger amount of eye contact and assertive behavior compared to OC users during the TSST. In this line, Troisi et al. (1999) [16] found a positive relationship between anxiety and eye contact, which seems to agree with the association found in our study. In addition, women in the follicular phase also displayed a slightly larger amount of flight, submission and displacement behaviors. In summary, it appears that women in an early follicular phase are psychologically more sensitive to stress, and this is also reflected in their non-verbal response.

Regardless group differences, we confirmed the hypothesis that the most common patterns of behavior displayed by women in response to an acute social stressor were flight and affiliation (see Table 2). These results point, on one hand, to the pattern of flight response described by Cannon (1932) [20] and, more importantly, to the tend-and-befriend strategy described by Taylor et al. (2000) [21]; in other words, women seem to adopt a gender specific strategy of behavioral coping, making a large use of patterns of affiliative behavior as an adaptive stress response.

The COPE scores provide information about the way an individual usually copes with stress situations. Although we did not expect to find dramatic differences between women groups, our study revealed that coping strategy was not fully the same between OC users and non-users. Indeed, follicular women obtained lower scores in the behavioral, cognitive and emotional dimensions of active coping compared to OC users. An interesting implication of this evidence is that the increasing use of oral contraceptives in young women and the associated differences in coping styles compared to non-users suggest that future studies should focus on this specific female population.

Although the available literature on acute physiological stress responsivity underscores the similarities between follicular women and OC users, our data point to undeniable differences in psychological and behavioral parameters. These differences could be explained by hypothesizing greater mood stability in OC users [38] and higher anxiety and negative feelings in follicular counterparts, as recently suggested by other authors [39], [40].

In our study we failed to find clear relationships between the most frequent categories of behavioral stress response (affiliation and flight) and concurrent psychophysiological changes for the group as a whole. However, in OC users group we found positive relationships between these categories and the basal levels and AUCg of cortisol, but negative ones with mood and anxiety reactivity. These results support the two common theories of stress from a biological perspective, that is, OC users showed the most typical stress reactions (flight and affiliation) when they have higher levels of cortisol. The hypothesized associations between heart rate values and cortisol levels with submissive behavior were confirmed, namely stress tachycardia and hypocortisolism were associated with high scores of submission. Moreover, baseline cortisol levels were negatively correlated with the amount of submission during the test: the lower the resting activity of the HPA axis the larger the use of submissive patterns of nonverbal behavior during the acute psychosocial challenge. In other words, a highly submissive strategy of coping with a stressor appears to be anticipated by a lower HPA axis activity. This evidence is in line with the general view that high cortisol levels prior to a challenge are functional to an active engagement with the stressful situation (physiological anticipation) [41], [42]; in the present study, low cortisol levels allowed only a submissive/passing strategy of coping with the TSST.

Another interesting outcome of this study was the negative relationship between the overall heart rate response to the stressor (AUC_G_) and displacement. This result agrees with a recent study in luteal women that found a negative relationship between the AUC_G_ of heart rate and displacement behavior [24]. These behavioral-autonomic associations seem to confirm the view on the dearousing properties of displacement behaviors [43] and suggest that they likely represent a successful behavioral strategy for promoting a prompter return to baseline homeostatic conditions. In addition, we also found that assertion (consisting of facial expressions and head movements that signal low-level aggression and hostility) and eye contact are linked to negative mood states. This result resembles what is reported in Troisi (1999) [16], who described a higher percentage of assertive behavior in depressed women compared to healthy counterparts.

When behavioral-physiological correlations where performed within each women group, findings were replicated only for women in the follicular phase. We can speculate that estrogens’ concentrations may be regulating the behavioral stress response. These results highlight the importance of controlling for the menstrual cycle phase and the use of oral contraceptives when studying the behavioral stress response with the neuroendocrine regulation jointly.

The finding that the active coping dimension of the COPE questionnaire correlated negatively with assertion and eye contact supports the relationships found between changes in mood and assertion and eye contact behaviors. It seems that women who usually face stressful situations with a less active coping strategy do also decreased positive mood and exhibit low-level aggressive behavior.

To the best of our knowledge, this is the first study that analyzed the behaviors displayed during and the psychophysiological responses to the TSST in two different groups of young, healthy women. Recently, two studies analyzed displacement behaviors during the TSST, but only in luteal women, or compared to men [23], [24]. This is the first time that OC users’ behavior analyzed during the TSST, together with their psychophysiological stress response and compared to women during the follicular phase with ECSI and COPE questionnaire. One could speculate that the use of oral contraceptives is influencing several factors of the stress response, modulating not only the physiological stress response (e.g. cortisol), but also the psychological (cognitive states and coping styles as a trait) as well as the behavioral stress reactions, i.e. the ability to face it.

In the present study, in spite of the relatively small sample size, differences in coping behavior between follicular women and oral contraceptives were found. We believe that these results, besides supporting previous findings in different groups of women, also provide relationships involving behavioral and physiological stress responsivity, such as the negative association between cortisol and submission. This finding looks important in view of a clearer definition of the biological substrates of active vs. passive coping strategies [18]. In addition, we wish to highlight the inverse relationships between the amount of displacement behavior during the TSST and heart rate responses on one hand and anxiety levels (obtained via questionnaires) on the other hand. They underscore the importance of distinguishing - and possibly combining in the same study- self reports with objectively measured behavioral and physiological responses.

### Limitations of this study

The results of this study are be interpreted with caution, due to the large number of correlations performed with a relatively small sample of subjects. The appropriateness of using multiple comparisons in such a situation is often debated, due to the increases of type I error. However, the choice of multiple comparisons corrections also increases the possibility of carrying type II error, thus hampering potentially important findings. Undoubtedly, future studies with larger samples are needed to further validate these results.

Another limitation of this study is that it did not include for comparison groups of women in other phases of the menstrual cycle. More so, future research should also control for the menstrual cycle phase of OC users when measuring coping styles, in order to perform a more reliable comparison with other groups of free cycling women.

## Supporting Information

S1 Data **Original dataset of the study.**
(XLSX)
